# Personality, Depression, Social Functioning, and Suicidal Behavior in Depressed Older Adult Inpatients

**DOI:** 10.1093/geroni/igaa057.1262

**Published:** 2020-12-16

**Authors:** Ira Yenko, Jennifer Ho, Helene Geramian, Wing Jin Mak, Hyunyoung Ellen Park, Avner Aronov, Dimitry Francois, Richard Zweig

**Affiliations:** 1 Yeshiva University Ferkauf Graduate School of Psychology, Mountain View, California, United States; 2 Yeshiva University Ferkauf Graduate School of Psychology, The Bronx, New York, United States; 3 Ferkauf Graduate School of Psychology, Yeshiva University, The Bronx, New York, United States; 4 Yeshiva University Ferkauf Graduate School of Psychology, Bronx, New York, United States; 5 Yeshiva University, Ferkauf Graduate School of Psychology, Brooklyn, New York, United States; 6 Weill Cornell Medicine, White Plains, New York, United States

## Abstract

Older adults are at higher risk for completed suicide. However, research in late-life suicide for high-risk populations remains a neglected topic, with some researchers suggesting that our knowledge of risk factors and risk conferral remains incomplete and insufficient in their predictive ability. Personality processes, in the context of interpersonal problems, have been associated with suicidal behavior, depression, and social functioning, but have rarely been evaluated in samples of older adults during periods of highest risk. This study examined factors underlying the relationship between personality processes, depression, social role functioning, and suicidal behavior in older adult inpatients. It also examined the examined the additive effect of personality processes, social adjustment, and depression on suicidal behavior. Depressed middle aged and older adult inpatients (N=52; Age M= 66.88, SD= 8.76) completed self-report measures of personality pathology (IIP-PD-25), depression (GDS-30), social functioning (SAS-SR), and recent suicidal behavior (SIB). Our research found that while interpersonal pathology was positively associated with depression (GDS-30, ß = .37, p = .006) and social functioning (SAS-SR, ß = .384, p = .003), it was not associated with suicidal behavior. The combined model of social functioning and depression displayed a trend toward significance, but neither variable was robust enough to emerge as an independent predictor of suicidal behavior. However, bivariate analyses found moderate effect sizes between depression or social functioning and suicidal behavior. Risk for suicidal behavior likely involves dynamic, complex, and interrelated relationships with clinical implications regarding assessment within this population.

